# Assessing the time use and payments of multipurpose community health workers for the various roles they play—a quantitative study of the Mitanin programme in India

**DOI:** 10.1186/s12913-022-08424-1

**Published:** 2022-08-10

**Authors:** Samir Garg, Mukesh Dewangan, Prabodh Nanda, Krishnendhu C, Ashu Sahu, Lalita Xalxo

**Affiliations:** State Health Resource Centre, Chhattisgarh, Raipur India

**Keywords:** Community health workers, CHW, Health volunteers, Multipurpose workers, Time motion study, Time use, Incentives, Payments, Mitanin, India

## Abstract

**Background:**

Community health workers (CHWs) are crucial human resources for health. While specialist CHWs focus on a single disease vertically, the generalist or multipurpose CHWs perform wider functions. The current study was aimed at examining the time multipurpose CHWs spend on performing their different roles. This can help in understanding the importance they attach to each role. Since CHWs in many developing countries are classified as part-time volunteers, this study also aimed to assess the adequacy of CHW payments in relation to their time use.

**Methods:**

The study covered a well-established CHW programme in India's Chhattisgarh state. It had 71,000 multipurpose part-time CHWs known as Mitanins. Data collection involved interviews with a representative sample of 660 rural and 406 urban Mitanins. A semi-structured tool was designed and field tested. It included 26 pre-coded activities of CHWs placed under their six purposes or roles. Prompting and triangulation were used during interviews to mitigate the possibility of over-reporting of work by CHWs. The recall period was of one week. Descriptive analysis included comparison of key indicators for rural and urban Mitanins. A multi-variate linear model was used to find the determinants of CHW time-use.

**Results:**

The rural and urban Mitanins respectively spent 25.3 and 34.8 h per week on their CHW work. Apart from location (urban), the total time spent was associated with size of population covered. The time-use was well balanced between roles of service-linkage, providing health education and curative care directly, COVID-19 related work and action on social determinants of health. More than half of their time-use was for unpaid tasks. Most of the cash-incentives were concentrated on service linkage role. The average payment earned by Mitanins was less than 60% of legal minimum wage.

**Conclusion:**

The time-use pattern of Mitanins was not dictated by cash-incentives and their solidarity with community seemed be a key motivator. To allow wide ranging CHW action like Mitanins, the population per CHW should be decided appropriately. The considerable time multipurpose CHWs spend on their work necessitates that developing countries develop policies to comply with World Health Organisation's recommendation to pay them fairly.

**Supplementary Information:**

The online version contains supplementary material available at 10.1186/s12913-022-08424-1.

## Background

Internationally, Community Health Workers (CHWs) are considered as one of the cornerstones of comprehensive primary health care [1]. CHWs play a crucial role in improving access and coverage of health services for under-served populations [2]. CHWs are also considered as agents for the wider social change necessary for improving health of people [3]. There is a growing recognition of CHWs as crucial human resources for health, especially in the low- and middle-income countries (LMICs) [4]. The last couple of decades have seen a global resurgence of CHW programmes [5].

According to the WHO, CHWs should be members of the communities they work for, should be selected by the communities and answerable to them and have shorter and informal training than professional health-workers [2]. The International Labour Organisation (ILO) has listed CHWs as a distinct occupational group.

The work profile of CHWs is enormously diverse across countries and it has evolved over time [6]. CHW roles and work arrangements vary broadly, from preventing disease to promoting access to services, from engaging in highly specific disease-related activities to supporting primary health care in general [7]. Considering the mind boggling diversity that exists in roles of CHWs in different health systems, researchers have found it useful to categorise them. They can be either generalist or specialist CHWs based on the range of functions they perform [8]. The specialist CHWs focus on specific health issues such as maternal and child health, HIV, malaria, and tuberculosis infections treatment of acute respiratory infections and adolescent health [2, 9]. Their work is narrowly or vertically focused on a single disease or a single cluster of diseases [4]. The generalist CHWs on the other hand, usually perform wide ranging functions which include preventive, promotive and curative services in maternal and child health, disease control, nutrition, and surveillance [2]. The generalist CHWs have also been referred by some as multipurpose CHWs [10].

There has been a renewed interest in generalist or multipurpose CHWs. Researchers have found that increasing the range of functions of CHWs is both feasible and useful in increasing productivity [10, 11]. The WHO has recommended to prefer multipurpose CHWs in contexts where they are expected to be the main frontline and are integrated into primary health care teams [4]. The amount of time such CHWs spend on their various roles is crucial to understand the importance they attach to each role. Though a few studies have examined the different roles of multipurpose CHWs globally, none have looked at the time they spend on their different roles.

Another important aspect is that CHWs in many LMIC contexts are classified as volunteers [2]. Volunteers are expected to be part-time. The total time they spend on their work as CHWs is important to understand how part-time they are. This can have implications for understanding the adequacy of their payments. The WHO has recommended that the remuneration of CHWs should be based on their workload [4]. The amount of time part-time CHWs put into their work reflects their workload as well as the time they are willing to commit for the purpose. The time commitment of CHWs vis-a-vis the time required for expected services is important to design the target population each CHW covers [4]. But there is a shortage of such information in LMIC contexts with scaled up programmes of part-time CHWs with multiple and wide-ranging roles.

The Mitanin programme in Chhattisgarh state of India offers an example of a scaled-up model of part-time CHWs [12]. These CHWs, known as Mitanins, fall in the generalist or multipurpose category in the broadest sense of the term [13]. Their role is not restricted to any particular disease or cluster of diseases. They are expected to work on wide ranging issues under preventive and promotive health in addition to detection and treatment of illnesses and making appropriate referrals. The programme has completed two decades in rural areas. It has 67,570 CHWs in rural areas covering a population of around 24 million. In addition, there are 3,771 CHWs working in urban slums with an experience of a decade who cover a population of around 2.5 million [13].

The Mitanin programme has received a fair amount of attention from global researchers. It has been studied as a case of a scaled up CHW programme [12, 14]. Researchers have analysed the work of Mitanins on many primary care issues including malaria, tuberculosis, maternal and newborn care [15–17]. Research has also focused on their inter-sector action for social determinants of health (SDOH) including malnutrition and gender based violence [13, 18, 19]. Mitanins have been recognised as a leading example of CHWs acting as socio-political actors demanding accountability from government and acting as agents of social change [3, 20].

As argued above, understanding the time use by part-time volunteer CHWs in scaled-up programmes in LMICs is a much needed area for research. Despite the attention Mitanins have received in research, no studies are available on how much time they spend on their work as CHWs and how that is distributed across their various roles. The current study is aimed at addressing the above gap. The specific objectives of the study were:

1. To find out the time Mitanins spend on their work and its determinants
2. To find out the distribution of time spent by Mitanins on each of their important roles
3. To map the incentivised and non-incentivised activities performed by Mitanins
4. To assess the adequacy of payments received by Mitanins in relation to the time they spend for CHW work
5. To compare the above aspects for Mitanins working in the rural and urban areas

## Methods and materials

### Study context

Chhattisgarh is one of the poorest states in India. It was carved out of a bigger state in 2000 and a large part of the state is under-developed [17]. Around three-fourth of its population lives in rural areas. The socially vulnerable groups of the scheduled tribes and scheduled castes constitute around 42% of the state's population. Parts of the state have difficult geography and 44% the state is under forest cover [17]. The state inherited a weak health system and poor health indices. The initiation of Mitanin programme was a response jointly composed by the government and civil society [12, 19, 21].

The selection of Mitanins was done by the local communities with each habitation selecting a Mitanin from among its women residents willing to take up the voluntary role [21]. There was no compulsory education criterion for selection [12]. In rural areas, the average population covered by a Mitanin in 2020 was around 330. In difficult geographies with lower population density, the average population per Mitanin could be even lower [17]. The programme was expanded to urban areas in 2012–13 and each slum selected their Mitanin [22]. The urban slums are densely populated and average population per Mitanin is around 700 [22].

The Mitanin programme is fully funded by government. It has a dedicated supportive supervision structure since it started [12, 21]. A modular design was followed for training the CHWs. The initial curriculum was of five modules spread over a total of 18 days [21]. Thereafter several additions have been done to the curriculum and there is an annual residential training of seven days that includes the refresher modules also [13]. The Mitanin programme was a key evidence for the national roll-out of CHWs known as ASHA [16]. Mitanins got recognised as a part of the ASHA programme in 2006 but the programme retained its basic design and many of its original characteristics [13].

The roles played by Mitanin CHWs: According to the state guidelines of the programme, Mitanins are expected to play the following four roles [13]:

1. Health education: CHWs are expected to provide health education to community, families and individuals. This is focused on the preventive and promotive aspects of health. This involves inter-personal communication for behavioural change through home visits and community meetings.
2. Direct delivery of curative care: CHWs are expected to identify and treat common simple illnesses at home or community level. This includes diarrhoea, malaria, skin infections, minor injuries, minor aches etc. They identify pneumonia in children and sickness in newborns and also treat them when referrals are not feasible.
3. Linkage with formal healthcare services: CHWs identify, refer and accompany individuals or families for facilitating their appropriate linkage with formal healthcare services. This includes facilitating immunisation, ante-natal care, family planning services and institutional deliveries. They identify presumptive cases of tuberculosis, leprosy and cataract and refer them for confirmation and required care. They follow-up on cases of tuberculosis and leprosy. They refer complicated cases of communicable diseases like diarrhoea, pneumonia or malaria. The role has expanded further with changing epidemiological needs and health policies. More recent inclusions under the service-linkage role is to facilitate screening and follow-up for hypertension, diabetes and mental illnesses.
4. Action on the social determinants of health (SDOH): CHWs organise the community and vulnerable groups for collective action on SDOH. It includes areas like drinking water, nutrition, food and social security programmes and opposing gender based violence. Organising community meetings is a key task for this purpose [13].

#### Payments to Mitanins

When the programme started in 2002, Mitanins were inducted as purely honorary volunteers and there were no payments involved [21]. When the national CHW programme ASHA started in 2006 and Mitanins became a part of it, task-based cash incentives were introduced [13]. Initially the incentives were for limited to promoting and accompanying institutional deliveries and sterilisation cases [19]. Gradually the list of incentivised tasks got expanded to cover linkage with more services including some home based activities like newborn care [23]. In order to allow a certain minimum amount to be earned by each CHW every month, a set of incentives were introduced in 2014 for 'routine' tasks i.e. regularly occurring activities like maintaining records and attending monthly meetings [23].

### Study design

The current study had a cross-sectional design. The study covered the CHWs working in rural and urban parts of Chhattisgarh state. It was an observational time motion study. Time motion study is a methodology in which the time duration of every activity of a subject is recorded to establish the workflow and ensure efficiency and effectiveness [24, 25]. There were three options available in how to conduct a time motion study. The first was to collect data through direct observation by an external observer shadowing the sample CHWs for a time-period. This method is labour intensive and difficult to do for a large sample. Its biggest limitation is that direct observation can induce improved or biased performance which is known as the Hawthorne effect [24]. The second option was of using self-reported logs or detailed written records. The main limitation of this option is the possibility of over-reporting performance [24]. We used the third option, i.e. interviewing the CHWs using a semi-structured questionnaire. It offered the advantage of allowing a two-way interaction [24]. It can help in clarification of any doubts. The interview method allowed this study to cover a sample large enough to be representative for the state-wide programme.

### Data collection tools

A semi-structured questionnaire was designed to collect information on the activity-wise time spent by a Mitanin in the preceding one week. The semi-structured tool was pre-tested in the field. It helped in devising a modified list of activities under each role of CHWs. After field testing, a pre-coded list of 26 activities was included in the tool while allowing for additional activities to be added if reported by a respondent Mitanin. For each reported activity, further data was collected on whether it involved a cash incentive or not.

The interview based method for time-motion study carries the risk of over-reporting but we mitigated that by using prompting while interviewing. This involved asking details of work done in terms of place where each task was done and the exact topics covered. During the interviews, the interviewers also inspected the records CHWs maintained. The respondents were informed that the information they provided was being triangulated using the records. The record used for triangulation was the prescribed register maintained by CHWs covering each individual/family provided services by them, including the incentivised as well as non-incentivised tasks. The recall period was kept as one week to minimise the recall bias while capturing the variation in tasks over this period. The prompting used during interviews also helped in reducing the chances of recall bias.

In addition to the designated four roles described earlier, Mitanins were expected to carry out a number of tasks related to the COVID-19 pandemic including promoting behavioural change, door to door visits for surveillance of relevant symptoms and monitoring the cases under home isolation. The data collection was done in November 2020 and the first wave of the pandemic was in progress in Chhattisgarh in that period [26]. The sixth and the last purpose of time use was for data collection, routine reporting about their work and other paper work of administrative nature. Attending training programmes is a seasonal activity for Mitanins [13]. There was no training going on at the time of data collection. The time spent by Mitanins on receiving training was therefore asked separately.

When a Mitanin reported covering more than one role through a single task, she was asked to divide the time spent in the different roles. This was sometimes needed for home-visits and community meetings. E.g., there was an instance of a home-visit of 40 min involving health education as well as treatment of an illness and the Mitanin divided it into 30 and 10 min for the two roles respectively. Similarly, a community meeting of 2 h covered health education as well as SDOH work and the Mitanin divided it as one hour for each role.

The other aspects covered in the semi-structured questionnaire were—socio-demographic characteristics of Mitanin, the incentive amount earned by Mitanin in the previous month and any cash expenditure incurred by Mitanin. The last aspect covered the costs for transport and other expenses incurred by Mitanins in the course of carrying out their CHW duties.

### Data collection

The survey was conducted by an independent body that provides technical support to the state's department of health. Two separate sets of interviewers were deployed to cover the rural and urban samples simultaneously. The interviewers were trained in use of the semi-structured tool. The data collection was monitored closely and reviewed frequently by the authors of this study.

### Sampling

The study had two samples—a) rural CHWs b) urban CHWs. The samples were designed so as to be representative of the rural and the urban CHWs in the state. The required sample size was calculated for estimating a single mean with confidence level of 95% and 1% precision. We assumed the population standard deviation as 10. The sample calculation was done using the sample size calculator of 'epitools' available on the web at https://epitools.ausvet.com.au/onemean. The required minimum sample size calculated was 385.

When we started the study, there was uncertainty whether the survey team will be able to cover enough CHWs from the remote rural areas as accessibility was poor during the COVID-19 pandemic. According to the sample size calculated, we needed a minimum of three CHW respondents per block for each of the 146 rural blocks in the state. To account for the possibility of low coverage in some areas, we increased the sample to five per block. At the end of the survey, our team for the rural areas was able to cover 660 CHWs with an equitable share from the remote areas. The share of remote administrative divisions of Sarguja and Bastar in the covered sample was similar to other regions of the state.

The respondents were selected in both samples through systematic random sampling. The state has a total of 146 rural blocks (administrative divisions). For the rural sample, from the list of CHWs of each of the 146 rural blocks, five CHWs were selected using systematic random sampling. Similarly, there are a total of 28 urban units in the state's urban CHW programme. From the list of each urban unit in the state, 15 CHWs were selected through systematic random sampling. The survey was able to cover 406 CHWs.

The actual sample size covered was adequate for the statistical analyses needed for this study.

### Data analysis

A list of activities carried out by Mitanins was prepared and divided into the paid (incentivised) and the unpaid categories. Each activity on which Mitanins reported spending time in the preceding week was also classified into the six purposes described earlier.

Descriptive analyses were performed using cross tabulations. The key indicators were compared descriptively for rural and urban CHWs. Confidence intervals at 95% were reported in parentheses for important indicators.

Multivariate linear regression was carried out to find out the determinants of amount of time Mitanins spent on their work. The total time spent weekly on CHW work was the outcome variable. The explanatory variables included in the model were—population covered by CHW, years of experience as CHW, age, educational qualification category, household size, marital status of CHW, the administrative/geographical division they worked in and the social group category CHWs belonged to. The list of study variables is available in Additional File S1. The survey data was analysed using STATA 15 software.

In order to assess the payments, the average amount earned by CHWs per hour of work was calculated. This rate was compared against the minimum wage declared by the state government.

### Ethics approval

Informed consent was obtained from all subjects or their legal guardians if the respondents were illiterate The identities of CHWs interviewed were kept confidential. The dataset was completely anonymised before analysis. Ethics approval was obtained from the Institutional Ethics Committee of the State Health Resource Centre, Chhattisgarh.

## Results

### Sample profile

The socio-demographic profile of the Mitanin CHWs in the urban and rural sample is given in Table 1. The average age of Mitanins in rural and urban areas was similar and most of them were married. A larger share of the rural Mitanins was working in tribal areas than the urban Mitanins. A larger share of rural Mitanins belonged to the vulnerable social groups of the scheduled tribes and scheduled castes. The average population covered per rural Mitanin was around half of the population per urban Mitanin. The rural Mitanins had longer experience as CHWs than the urban Mitanins.

**Table 1:** Sample profile of Mitanin CHWs in rural and urban Chhattisgarh (2020)

Characteristic		Rural Mitanins (% with CI) (*N* = 660)	Urban Mitanins (% with CI) (*N* = 406)
Age of Mitanin	Mean	38.5 (38–39)	38.9 (38–40)
	Median	38 (37–39)	38 (37–39)
Household size	Mean	5.6 (5.4–5.7)	5.1 (4.9–5.3)
	Median	5 (5–5)	5 (4–5)
Years of experience as CHW	Mean	12.9 (12.6–13.3)	7.8 (7.6–7.9)
	Median	15 (14–16)	8 (8–8)
Population covered per CHW	Mean	361 (341–381)	748 (719–777)
	Median	305 (300–325)	704 (650–742)
Administrative/geographical Division	Raipur	17.9	40.2
	Durg	17.4	27.8
	Bilaspur	23.6	18.7
	Sarguja	23.2	7.6
	Bastar	17.9	5.7
Type of area	Tribal	43.0	79.3
	Non-tribal	57.0	20.7
Marital status	Married	98.8	98.0
	Unmarried	1.2	2.0
Social group (caste category)	Scheduled Tribes/ Scheduled Castes (ST/SC)	50.9	22.9
	Other Backward Classes (OBC)	43.2	59.9
	Others (General)	5.9	17.2
Education	8^th^ or higher standard	78.2	88.7
	5^th^ to 7^th^ standard	12.9	7.2
	1^st^ to 4^th^ standard	3.2	3.4
	No formal education	5.7	0.7

The findings have been presented here according to the study objectives as listed in the background. There is a section addressing each of the first four objectives. The findings on the fifth objective, of comparing urban and rural CHWs, have been presented alongside the findings on the first four objectives.

## Time spent by Mitanins on CHW work and its determinants

The amount of time spent by Mitanins on their work as CHWs in a week is given in Table 2 (in hours). The rural Mitanins spent an average of 24.1 h per week on CHW work. On an average, the urban Mitanins spent 9.5 h more time per week than the rural Mitanins.

**Table 2:** Hours spent by Mitanins in a week on different roles/purposes

Purpose/Role	Mean hours spent by Mitanins per week with 95% CI (*N* = 660)			
	**Rural**		**Urban**	
	**Hours**	**Share**	**Hours**	**Share**
Health Education	4.3 (4.1–4.6)	17.8%	5.0 (4.7–5.3)	14.9%
Detection of illness and treatment directly by Mitanin	2.1 (1.9–2.2)	8.7%	1.6 (1.5–1.8)	4.8%
Linkage with services	5 (4.6–5.5)	20.7%	13.5 (12.9–14.2)	40.2%
Action on Social Determinants of Health (SDOH)	2.9 (2.6–3.2)	12.0%	2.8 (2.5–3.1)	8.3%
COVID-19 related duties	3.7 (3.4–4)	15.4%	3.8 (3.5–4.1)	11.3%
Data Collection and reporting	6.0 (5.5–6.5)	24.9%	6.9 (6.3–7.5)	20.5%
**Total**	**24.1 (23.2–25)**	**100%**	**33.6 (32.5–34.7)**	**100.0%**

The mean no. of days for which the rural Mitanins received training in 2020 was 8.8 days. The average for the urban Mitanins was also 8.8 days of training. At seven hours per day of training, Mitanins spent 62 h in a year on training. It translated into around 1.2 h per week on training. After adding this component, the time spent by Mitanins per week became 25.3 h in rural and 34.8 h in urban areas.

The results of multivariate linear regression for finding the determinants of the total weekly time spent by Mitanins are given in Additional File S2. It showed that the weekly time spent was likely to rise with increase in population covered by a rural CHW. The time use was not associated significantly with time use of the urban CHWs. Both the rural and urban CHWs in one geographical division (Bilaspur) were likely to spend less time than the other divisions. None of the other variables were associated significantly with the weekly time use of the rural or urban CHWs.

## Distribution of time spent on different roles

The distribution of time use of rural Mitanins across the six purposes showed that around one-fourth time was spent on data collection, recording and reporting (Table 2). In terms of services, the community based work of health education and other direct services (detection and treatment of illnesses) by Mitanin accounted for 26.5% with a larger share belonging to the health education role. The service linkage role also occupied a significant share at 20.7%.

Compared to the rural Mitanins, the urban Mitanins spent around 8.5 h more on linkages with services. The linkage role accounted for 40.2% of the total time put in by the urban Mitanins. The hours spent for the other five purposes were quite similar for the rural and urban Mitanins.

## Mapping of paid and unpaid activities performed by Mitanins

The paid and the unpaid tasks performed by the Mitanins are listed in Table 3. In this list, the above activities have been divided across the different purposes/roles.

**Table 3:** Paid and unpaid activities performed by Mitanins

Purpose	Paid activities	Unpaid activities
Health Education	Home visits for health education—newborn care and young child care (0–15 month age children)	Home visits for health education—young child care (16–35 month children)
		Home visits for health education—pregnant women
		Home visits for follow-up of chronic disease cases—e.g. hypertension, diabetes, mental disorders, blood disorders
		Home visits for prevention of vector-borne and water-borne diseases e.g. dengue, malaria, diarrhoea
		School health meetings with students
		Meetings in community for health education on a wide range of health issues
Delivering services directly	Testing and treatment of malaria	Identification and treatment of illnesses at Mitanin's home or by visiting families—diarrhoea, cold and cough in children, pneumonia in children, sick newborn, skin ailments, pain, minor injuries, reproductive-tract infections, eye infection
		Contraceptive distribution and counselling
Linkage with formal services	Attending monthly immunisation session/village health and nutrition day (classified as a monthly routine task) and bringing children for immunisation	
	Bringing pregnant women to local ante-natal care session,	Identification of high risk pregnancies, taking pregnant women to secondary care facilities for ante-natal check-ups, diagnostics and management
	Identification of referrals and accompanying them to health facilities—deliveries, sterilisation, cataract, severely malnourished children	Identification of referrals or accompanying them to higher health facilities—sick newborn, pneumonia, non-communicable diseases, mental illnesses, injuries or other illnesses
	Identification of presumptive cases of tuberculosis, leprosy; referring them to health facilities for confirmation; follow-ups for treatment adherence	Referrals for IUCD (other than PPIUCD)
	Specific campaigns for linkage with services—pulse polio, de-worming, filaria prophylaxis	Campaign for Vitamin A supplementation
	Bringing patients especially above 30 years age individuals to health and wellness centres for screening of non-communicable diseases	
Action on social determinants of health (SDOH)	One monthly meeting of village health nutrition sanitation committee—for action on social determinants (classified as a monthly routine task)	Other community meetings for action on social determinants
		Visits to officials of non-health sectors (water, food, employment etc.)
		Home visits, community meetings and action on opposing gender based violence
COVID-19 related action^a^		Home visits for COVID-19 related tasks-door to door survey to find persons with influenza symptoms, monitoring home isolation
		Bringing individuals for testing, bringing individuals for COVID-19vaccination
		Data collection and reporting related to COVID-19
Recording and reporting	Collecting data on population, symptoms and risk factors of multiple diseases in individuals using community based assessment checklist	Other surveys asked by government health officials
	One monthly meeting for reporting to supervisors (classified as a monthly routine task)	Other meetings for reporting or for receiving instructions about activities for specific government campaigns and other priorities
	Recording data in CHW register and updating monthly (classified as a monthly routine task)	

^a^Though a monthly cash incentive was announced by the central government for the work specific to COVID-19 pandemic, it was not paid to Mitanins. We have therefore placed this work under unpaid activities

The detailed activity-wise distribution of time spent by Mitanins is given in Additional File S3. Based on the above, proportion of time spent on unpaid work under each role is summarised in Table 4.

**Table 4:** Proportion of time use belonging to tasks that were unpaid

Role/Purpose	Proportion of time use on unpaid tasks (%)	
	**Rural Mitanins (*n* = 660)**	**Urban Mitanins (*n* = 406)**
Health Education	64%	62%
Detection of illness and treatment directly by Mitanin	76%	96%
Linkage with services	16%	34%
Action on Social Determinants of Health (SDOH)	65%	64%
COVID-19 related duties	100%	100%
Data Collection and reporting	40%	38%
**Total**	**55%**	**52%**

Table 4 showed that rural and urban Mitanins respectively spent 55 and 52% of their time on unpaid tasks. A large share of the time spent on health education, direct curative services, SDOH and COVID-19 related roles was unpaid. A large share of the time spent on roles of service linkage, data collection and reporting involved payments (Additional File S3).

## Assessment of payments received by Mitanin CHWs

The mean monthly payment of rural and urban Mitanins for the month of November 2020 was found to be Indian Rupees (INR) 3980 (3738–4223) and INR 4932 (4630–5265) respectively.

Mitanins incurred expenses out of their pocket in the course of fulfilling their tasks as CHWs. The mean expenditure was found to be INR 269 per month in rural and INR 240 per month in urban areas (Table 5). A major share of this expenditure was on transportation while accompanying patients to health facilities.

**Table 5:** Mean out of pocket expenditure incurred by Mitanins in a month (in INR)

Purpose	Mean out of pocket expenditure incurred by Mitanins in month preceding the survey (in INR)	
	**Rural (*N* = 660)**	**Urban (*N* = 406)**
Phone	17	30
Photocopying	11	16
Travel for accompanying patients to health facilities	156	132
Travel for meetings	31	24
Other expenses	54	38
**Total**	**269**	**240**

The net average payment after deducting the above expenses was INR 3711 per Mitanin in rural and INR 4692 in urban areas. The mean payment of INR 3711 for rural Mitanins translated into INR 866 per week. For 25.3 h of work per week, the average hourly payment was INR 34 per hour for rural Mitanins. The minimum wage declared by government for skilled workers in rural areas was INR 400 per day in 2020 which translates into an hourly rate of INR 57 [27]. This showed that the average payment to Mitanins in rural areas was 60% of the legal minimum wage.

For urban Mitanins, the mean payment of INR 4692 translated into INR 1095 per week. For 34.8 h of work per week, the average hourly payment was INR 32 per hour for urban Mitanins. The minimum wage declared by government for skilled workers in urban areas was INR 420 per day which translates into an hourly rate of INR 60 [27]. The average payment to Mitanins in urban areas was 53% of the legal minimum wage.

## Discussion

As the importance of CHWs gets better recognised by health systems globally, the debates on their role, time-use and payments have also grown. Also, as the need grows to integrate CHWs into primary care systems in LMICs, it will demand moving towards the multipurpose role-design. This study is among the first to examine the time spent by multipurpose and part-time CHWs on their different roles in a LMIC context. The study was conducted in the context of Mitanin CHWs, a scaled-up and government funded CHW programme in operation for around two decades in an under-developed province of India. The following discussion on our findings and their implications is arranged according to the study objectives.

### Overall time-use of CHWs

The current study found that Mitanins in rural areas were spending 25.3 h per week on their work as CHWs whereas their urban counterparts put in 34.8 h per week. In comparison, the full-time employees of government in the state were expected to work for 35 h a week [28]. This shows that though Mitanin CHWs were classified as part-time volunteers, the time they spent for their work was approaching the full-time level.

An evaluation of ASHA in rural areas of multiple states in 2010–11 showed that they were spending more than 15 h per week, reaching up to 25 h in some states [29]. More recent studies on rural ASHA have reported that they put in 20 to 28 h per week [25, 30–33]. While comparing the hours put in ASHA and Mitanin, it is important to keep in mind the average size of population they cover. While the average population covered per rural Mitanin was 361, it was around three times greater for the ASHA in other states [23]. While the population covered per Mitanin differs from ASHA, both put in a similar amount of time per week. Studies show that Mitanins have been effective in multiple aspects of health whereas evidence has been less pronounced in case of ASHA [14, 15, 17, 34]. Studies have also shown that Mitanins have more intensive contact than ASHA [16, 17]. This shows that if a similar intensity of contact and range of activities is to be achieved by ASHA, the average population per ASHA needs to be brought down by increasing the number of ASHAs deployed nationally.

How much time should CHWs spend on their work? A study on CHWs in Zanzibar found that spending 18 h a week was adequate to achieve desired contact with a population of 700 [35]. In Rwanda, a study concluded that weekly 23 h were needed to cover a population of 160 [35]. Comparing the time-use pattern in different CHW programmes can pose difficulty as apart from varying population size; the expected roles, activities and intensity of coverage vary widely.

### Distribution of time-use across different roles

The current study also suggests that to allow for work on a range of roles including SDOH, the population per CHW needs to be decided accordingly. Among the various purposes; data collection, recording and reporting occupied around one-fourth time of Mitanins. Existing studies of ASHA have reported a even higher proportion of time being spent on such activities [25, 30, 32, 36]. There is a need to recognise such high allocation of time to administrative tasks as a problem because it can reduce the time available with CHWs for actual delivery of services.

### Mapping of paid and unpaid tasks of CHWs

The mapping of CHW activities in this study helped in understanding the variety of tasks they performed within each role. This suggests that understanding and comparing multipurpose CHW programmes requires unpacking the activities they carry out for their various roles.

Governments tend to emphasise the service-linkage role in the work of CHWs [37]. They also tend to undermine the work on SDOH and the direct services by CHWs [13]. The incentive structure designed in India is also heavily tilted towards the service-linkage role [37]. Though cash incentives have got introduced for some types of home visits (for improving infant care practices) and direct services (detection and treatment of malaria), there are hardly any incentives for work on other direct services or action on SDOH [23]. The current study showed that Mitanins were able to spend a considerable amount of time on SDOH. Mitanins spent a significant share of time on providing services at community level in form of health education and direct curative care. Some studies on ASHA have indicated that the incentive structure has played a central role in shaping their work as CHWs [36, 38]. The current study found that Mitanins spent a significant time on working for SDOH, health education and direct services despite the lack of cash incentives for such work. This suggests that a feeling of solidarity with the community could be a key source of motivation for the Mitanins. This shows that there are factors beyond the cash incentives that influence CHW motivation.

Existing research on ASHA has shown the competing demands on her time from the stakeholders she feels accountable to [37]. Their families expected them to earn for household while continuing to perform household chores [37]. The formal health authorities expected them to give more and more of their time for the service linkage role [13, 37]. The local communities wanted CHWs to take care of common illnesses [13, 37]. In the current study, Mitanins spent a considerable amount of time on their work as CHWs and more than half of the time use was on unpaid tasks. The amount of time they spent on the unpaid tasks that directly benefit the community shows the solidarity they feel with their community. Researchers have concluded that ASHA CHWs need to be seen as skilled social actors who are able to balance the conflicting demands on their time [37]. The time-use pattern found in the current study could also be a reflection of Mitanins acting as skilled social actors. Another factor helping Mitanins in achieving this balance could be the mutually reinforcing nature of their different roles. Different roles, while placing competing demands on their time, were still mutually supportive. E.g., a Mitanin excelling in providing or linking with services can earn high credibility in the community and that in turn can help her in acting as a leader for work on SDOH [13].

Researchers have expressed that CHWs can play an important role in pandemics in work related to contact tracing, community engagement, fighting stigma and SDOH [39, 40]. They have also advocated for governments to provide better support to CHWs [40]. The current study sheds some light on the work of CHWs during the COVID-19 pandemic. It found that the Mitanin CHWs were working actively during the pandemic, attending to their multiple roles. This shows that CHWs played an important part in maintaining some of the essential healthcare services during the COVID-19 pandemic. It also found that there were additional duties they performed which were directly related to the pandemic.

### Assessment of payments received by CHWs

According to the design of Mitanin programme at its beginning (in 2002), a Mitanin was expected to put in 8 to 10 h per week [21]. This design was aimed at keeping the workload in line with Mitanins being purely honorary volunteers receiving no payments at all. Most of the Mitanins at that time were engaged in the farming and forest produce collection work of their families. The premise was that the work burden as CHWs should be small enough to allow them to continue with their existing livelihoods [21]. When the ASHA programme started in 2006, the expectation from them was to put in 15 h per week for CHW work [29]. When Mitanins became a part of the national CHW programme in 2006, cash incentives got introduced [41]. These incentives were mainly for promoting institutional deliveries and sterilisations and involved small payments. From 2007 to 2011, Mitanins had an average earning of around INR 200 per month [41]. Gradually more tasks were added and incentives were also expanded. The current study found that in 2020, Mitanins spent three times the hours than what was expected at beginning of the programme. Being a CHW seems to be their main occupation now with little possibility of continuing their traditional livelihoods. They have gained substantial experience and skills. Yet, their net earnings from CHW work fall 40% short of the legal minimum wage of skilled workers in the state. This shows the need for government to increase their payments to at least match the legal minimum wage. CHWs in Ethiopia were also putting in a similar amount of time and being paid poorly [42]. A consensus has started to emerge that CHWs need to be paid [43]. The WHO has also recommended that CHWs should be paid adequately by taking into account their workload and its complexity [4]. Examples are now available of programmes that fulfil the above recommendation from WHO e.g. Brazil and Nigeria [43]. Like the study in Ethiopia, the current study also showed that CHWs incur significant out of pocket expenses in the course of their duties [42]. There is a need to take into account such payments while assessing earnings of CHWs.

The failure of the government to pay for the COVID-19 pandemic related work of Mitanins was found to be a crucial gap. This suggests the need for governments to pay the CHWs adequately while engaging them in additional duties during pandemics and other emergencies.

CHW remuneration and payment mechanisms differ across countries based on the purpose of programme, government's commitment and availability of funds [34]. The female community health volunteers in Nepal are an example of unpaid CHWs. There are CHW programmes with fixed payments such as the Behvarz in Iran and the lady health workers in Pakistan [34]. The ASHA programme in India has been seen as an example of a payment design relying solely on task based cash incentives [34, 38]. The same design was followed in case of the Mitanin CHWs. Though the inclusion of some routine tasks in the incentive structure has allowed ASHA and Mitanins to earn a minimum assured income every month but these are still task-based incentives and not a fixed payment. Some studies have cautioned against a payment design exclusively based on incentives [44]. The guidance from WHO is to include a fixed component in CHW remuneration [4]. However, a fixed payment may also carry the danger of limiting the autonomy of CHWs to decide how much they want to work and when [13]. It may also bring them under pressure to adhere to government priorities as opposed to the needs of their communities [13]. One option can be to increase the remuneration of Mitanins without necessarily changing the existing payment arrangements. CHWs need to be paid fairly for their labour but the payment mechanism should be designed to support their autonomy in deciding work priorities according to the community's health needs.

The current study also provided a comparison of time-use for the rural and urban CHWs. The urban Mitanins spent more time on CHW work than the rural Mitanins. The difference was explained largely by the additional time urban Mitanins spent on linkage with services. This could be related to greater availability of healthcare services in urban areas. The size of population a rural Mitanin looked after was found to be the main determinant of weekly time use. This suggests that total time put in by CHWs is influenced by population as well as contextual factors like the geographical availability of formal healthcare services.

In terms of methods, the current study offers a feasible approach to assess the time use of CHWs. The key strengths of this study were that it found out the time use for each activity and role of CHWs and it had an adequately large sample size. The mapping of paid and unpaid tasks done at different points of time can provide valuable information on how a programme has evolved over time. Further research will be needed to identify the sources of motivation underlying the time use pattern and qualitative methods may be more suitable for that. Further research is recommended in form of case studies of different CHW programmes in LMIC contexts to understand the interactions between the time use for multipurpose CHWs and the sources of their motivation.

### Limitations

Though significant measures were taken to address the possibility of over-reporting, the method may still carry some limitations of self-reporting of work. The recall period used of one week has limitation of not taking into account any seasonality in CHW activities but we do not expect seasonality to alter the pattern of our findings. The time use of CHWs can be influenced by multiple factors including their training, kind of supportive supervision and availability of necessary inputs (e.g. medicines) but the current study could not delve into these aspects [25].

## Conclusions

Considering the amount of time Mitanin CHWs spend on their work, it will be a mistake to continue to visualise them as part-time and honorary volunteers. There is a clear case for increasing their payments. Yet, the time-use of Mitanins was not dictated by the structure of cash incentives. Their time-use was well distributed among their various roles as multipurpose CHWs. To allow CHW action in wide ranging roles to be meaningful, the population per CHW needs to be decided appropriately. 

## Supplementary Information

**Additional file 1.**

**Additional file 2.**

**Additional file 3.**

## Data Availability

The datasets used and/or analyzed during the current study are available from the corresponding author and State Health Resource Centre, Chhattisgarh on reasonable request.

## Abbreviations

ASHA: Accredited Social Health Activists
CHWs: Community Health Workers
CI: Confidence Interval
LMICs: Low- and Middle-Income Countries
OBC: Other Backward Classes
SC: Scheduled Castes
ST: Scheduled Tribes
SDOH: Social Determinants of Health
WHO: World Health Organisation

## References

[1] World Health Organisation. Declaration of Alma-Ata International Conference on Primary Health Care, Alma-Ata, USSR, 6–12 September 1978. Available at: https://cdn.who.int/media/docs/default-source/documents/almaata-declaration-en.pdf?sfvrsn=7b3c2167_2. Accessed on 12 Mar 2022

[2] Lehmann U, Sanders D, Community health workers: What do we know about them (2007). The State of the Evidence on Programmes, Activities, Costs and Impact on Health Outcomes of Using Community Health Workers.

[3] Schaaf M, Fox J, Topp SM, Warthin C, Freedman LP, Sullivan R (2018). Community health workers and accountability: reflections from an international “think-in”. Int J Equity Health.

[4] World Health Organisation (2018). WHO guideline on health policy and system support to optimize community health worker programmes.

[5] Schneider H, Okello D, Lehmann U (2016). The global pendulum swing towards community health workers in low- and middle-income countries: a scoping review of trends, geographical distribution and programmatic orientations, 2005 to 2014. Hum Resour Health.

[6] Schneider H (2019). The governance of national community health worker programmes in low- and middle-income countries: an empirically based framework of governance principles, purposes and tasks. Int J Health Policy Manag.

[7] Perry HB, Zulliger R, Rogers MM (2014). Community health workers in low-, middle-, and high-income countries: an overview of their history, recent evolution, and current effectiveness. Annu Rev Public Health.

[8] Love MB, Gardner K, Legion V (1997). Community health workers: who they are and what they do. Health Educ Behav.

[9] Koon AD, Goudge J, Norris SA (2011). A review of generalist and specialist community health workers for delivering adolescent health services in sub-Saharan Africa. Hum Resour Health.

[10] Shelley KD, Frumence G, Mpembeni R, George AS, Stuart EA, Killewo J (2018). Can volunteer community health workers manage multiple roles? An interrupted timeseries analysis of combined HIV and maternal and child health promotion in Iringa, Tanzania. Health Policy Plan.

[11] Bezbaruah S, Wallace P, Zakoji M, Perera WLSP, Kato M (2021). Roles of community health workers in advancing health security and resilient health systems: Emerging lessons from the COVID-19 response in the South-East Asia Region. WHO South-East Asia J Public Health.

[12] Nambiar D, Sheikh K (2016). How a technical agency helped scale up a community health worker program: an exploratory study in Chhattisgarh state. India Health Systems & Reform.

[13] Garg S and Pande S. Learning to Sustain Change: Mitanin Community Health Workers Promote Public Accountability in India. Accountability Research Center, 2018. Accountability Note 4. Washington DC. Available at: www.accountabilityresearch.org/publications. Accessed on 12 Mar 2022

[14] Sundararaman T (2007). Community Health-workers: scaling up programmes. The Lancet.

[15] Vir S, Kalita A, Mondal S, Malik R (2014). Impact of community based Mitanin program on undernutrition in rural Chhattisgarh state. Food Nutr Bull.

[16] Bajpai N, Dholakia RH. Improving the Performance of Accredited Social Health Activists in India. 2011. Working Paper No. 1 May 2011. Columbia Global Centers. South Asia, Columbia University. Mumbai. 10.7916/D8988G63. Accessed on 12 Mar 2022

[17] Garg S, Gurung P, Dewangan M, Nanda P (2020). Coverage of community case management for malaria through CHWs: a quantitative assessment using primary household surveys of high-burden areas in Chhattisgarh state of India. Malar J.

[18] Nandi S, Schneider H (2014). Addressing the social determinants of health: a case study from the Mitanin (Community Health Worker) programme in India. Health Policy Plan.

[19] Champa A. Enabling Social Accountability: The Community Health Worker Programmes of Chhattisgarh and Jharkhand.”Policy Report No.21. 2017 Available at: http://www.thehinducentre.com/publications/policy-report/article9694936.ece. Accessed on: 12 Mar 2022

[20] Scott K, George AS, Ved RR (2019). Taking stock of 10 years of published research on the ASHA programme: examining India’s national community health worker programme from a health systems perspective. Health Research Policy and Systems.

[21] State Health Resource Centre (2002). Mitanin Programme: Conceptual Issues and Operational Guidelines, SHRC.

[22] Garg S, Khewar A, Rizu K (2016). Improving access to health in urban slums through rollout of NUHM and expansion of community processes: the experience of Chhattisgarh. BMJ Global Health.

[23] National Health Systems Resource Centre. Update on ASHA programme - July 2019. New Delhi. 2019. https://nhsrcindia.org/sites/default/files/2021-06/ASHA%20Update%20July%202019.pdf Accessed on: 12 Mar 2022

[24] Lopetegui M, Yen PY, Lai A, Jeffries J, Embi P, Payne P (2014). Time motion studies in healthcare: what are we talking about?. J Biomed Inform.

[25] Chebolu-Subramanian V, Sule N, Sharma R, Mistry N (2019). A time motion study of community mental health workers in rural India. BMC Health Serv Res.

[26] Garg S, Bebarta KK, Tripathi N, Krishnendhu C (2022). Catastrophic health expenditure due to hospitalisation for COVID-19 treatment in India: findings from a primary survey. BMC Res Notes.

[27] Government of Chhattisgarh. Minimum daily wages. Collector of Raipur. Date 28th October 2020. Available at: https://raipur.gov.in/notice_category/announcements/ Accessed on: 12 Mar 2022

[28] Government of Chhattisgarh. Change in timings of work in government offices. General Administration Department. Date 1st February 2022. Available at: https://www.gad.cg.gov.in/notice_display.aspx Accessed on: 12 Mar 2022

[29] National Health Systems Resource Centre (2011). ASHA Which way forward?.

[30] Kawade A, Gore M, Lele P, Chavan U, Pinnock H, Smith P, for the RESPIRE collaboration (2021). Interplaying role of healthcare activist and homemaker: a mixed-methods exploration of the workload of community health workers (Accredited Social Health Activists) in India. Hum Resour Health.

[31] Oswal K, Kanodia R, Pradhan A, Avhad M, Sethuraman L, Kharodia N (2020). The role of frontline community health workers in the non-communicable disease screening program in Assam, India: current trends, challenges and scope - A time and motion study. J Cancer Policy.

[32] Surekha A, Suguna A (2019). Time Motion Study for the Effectiveness of Intervention by Accredited Social Health Activists (ASHAs) in the control of hypertension and diabetes in a rural population of Kolar District, Karnataka. Nat J Res Community Med.

[33] Mahalingashetty, A. Work-time analysis of ANM and ASHA: A Priority for Strengthening Health Systems. Columbia University. 2012. Available at: https://aditigondal.files.wordpress.com/2012/08/anuraga-mahalingashetty-capstone-may-2012.pdf. Accessed on: 12 Mar 2022.

[34] Singh D, Negin J, Otim M, Orach CG, Cumming R (2015). The effect of payment and incentives on motivation and focus of community health workers: five case studies from low- and middle-income countries. Hum Resour Health.

[35] Morrow M, Sarriot E, Nelson AR, Sayinzoga F, Mukamana B, Kayitare E (2021). Applying the community health worker coverage and capacity tool for time-use modeling for program planning in Rwanda and Zanzibar. Glob Health Sci Pract.

[36] Guha I, Raut A, Maliye CH, Mahendale AM, Garg B. Qualitative Assessment of Accredited Social Health Activists (ASHA) Regarding their roles and responsibilities and factors influencing their performance in selected villages of Wardha. Int J Adv Med Health Res. 2018;5(1):21–26.

[37] Kane S, Radkar A, Gadgil M, McPake B (2021). Community health workers as influential health system actors and not “Just Another Pair Of Hands”. Int J Health Policy Manag.

[38] Scott K, Shanker S (2010). Tying their hands? Institutional obstacles to the success of the ASHA community health worker programme in rural north India. AIDS Care.

[39] Bhaumik S, Moola S, Tyagi J (2020). Community health workers for pandemic response: a rapid evidence synthesis. BMJ Glob Health.

[40] Patricia JP, Islam N, Matiz LA (2020). Community health workers and covid-19 — addressing social determinants of health in times of crisis and beyond. N Engl J Med.

[41] Misra JP. Evaluation of the Community Health Volunteer (Mitanin) Programme. European Union State Partnership Programme. 2011. Available at: http://health.cg.gov.in/ehealth/MitaninFinalReport11thMarch2011.pdf. Accessed on: 10 Mar 2022

[42] Ethiopian Federal Ministry of Health. Health Extension Workers Time Motion Study Complemented by In-depth Interviews within Primary Health Care Units in Ethiopia (2015). FMOH, HEPCAPS II Project.

[43] Ballard M, Westgate C, Alban R, Choudhury N, Adamjee R, Schwarz R (2021). Compensation models for community health workers: Comparison of legal frameworks across five countries. J Glob Health.

[44] Bhatia K. Performance-based incentives of the ASHA scheme stakeholders’ perspectives. Econ Pol Wkly. 2014;49(22):145–51.

