# Supplementary material for: Assessing the time use and payments of multipurpose community health workers for the various roles they play—a quantitative study of the Mitanin programme in India
Source: BMC Health Serv Res. 2022 Aug 10;22:1018. doi: 10.1186/s12913-022-08424-1 (PMC9364297; doi:10.1186/s12913-022-08424-1)
Supplement: Supplementary file 1 — Additional file 1. [file 12913_2022_8424_MOESM1_ESM.docx]

|  | |  | |  | | |  | |
| --- | --- | --- | --- | --- | --- | --- | --- | --- |
|  | | | **Additional File S1**  **Table: List of variables included in the study** | | | | |  |
| **Variables** | **Description** | | | | **Type of variable** | **Category** | |  |
| Time use | Hours spent by a CHW on her work as a CHW in a week | | | | Continuous |  | |  |
| Population | Population covered by a CHW | | | | Continuous |  | |  |
| Age | Age of CHW (years completed) | | | | Continuous |  | |  |
| Experience | Years of experience in CHW role (years completed) | | | | Continuous |  | |  |
| Household size | Size of CHW's household | | | | Continuous |  | |  |
| Social Group | Social Group/Caste of CHW | | | | Categorical | Scheduled Tribe (ST) | |  |
|  |  |  |  |  |  | Scheduled Caste (SC) | |  |
|  |  |  |  |  |  | Other Backward Classes (OBC) | |  |
|  |  |  |  |  |  | Others | |  |
| Education | Education qualification Category of CHW | | | | Categorical | 8th standard or higher | |  |
|  |  |  |  |  |  | 5-7th standard | |  |
|  |  |  |  |  |  | 1-4th standard | |  |
|  |  |  |  |  |  | No formal education | |  |
| Administrative division | Administrative divisions of Chhattisgarh state where CHW works | | | | Categorical | Raipur | |  |
|  |  |  |  |  |  | Bilaspur | |  |
|  |  |  |  |  |  | Durg | |  |
|  |  |  |  |  |  | Sarguja | |  |
|  |  |  |  |  |  | Bastar | |  |
| Marital Status | Marital status of CHW | | | | Categorical | Currently Married | |  |
|  |  |  |  |  |  | Currently not married | |  |
