# Supplementary material for: Assessing the time use and payments of multipurpose community health workers for the various roles they play—a quantitative study of the Mitanin programme in India
Source: BMC Health Serv Res. 2022 Aug 10;22:1018. doi: 10.1186/s12913-022-08424-1 (PMC9364297; doi:10.1186/s12913-022-08424-1)
Supplement: Supplementary file 2 — Additional file 2. [file 12913_2022_8424_MOESM2_ESM.docx]

**Additional File S2**

**Table: Linear regression models for determinants of total hours work by Mitanin in previous week for rural and urban areas**

|  | **Rural** | | | | **Urban** | | | |
| --- | --- | --- | --- | --- | --- | --- | --- | --- |
|  |  |  | Number of obs | 660 |  |  | Number of obs | 406 |
| **Time use (hours per week)** |  |  | R-squared | 0.12 |  |  | R-squared | 0.16 |
|  | **Coef.** | **P>t** | **[95% Conf. Interval]** | | **Coef.** | **P>t** | **[95% Conf. Interval]** | |
| **Population covered by CHW** | 0.004 | 0.046* | 0.0001 | 0.007 | 0.001 | 0.478 | -0.002 | 0.005 |
| **Experience as CHW (years)** | 0.107 | 0.370 | -0.127 | 0.342 | 0.206 | 0.572 | -0.509 | 0.922 |
| **Geographical/ Administrative Division ((Reference category - Raipur)** |  |  |  |  |  |  |  |  |
| Durg | -0.915 | 0.555 | -3.958 | 2.127 | 0.099 | 0.944 | -2.677 | 2.875 |
| Bilaspur | -3.110 | 0.033* | -5.960 | -0.260 | 4.231 | 0.008* | 1.089 | 7.372 |
| Sarguja | -2.436 | 0.109 | -5.415 | 0.542 | 3.550 | 0.118 | -0.899 | 7.998 |
| Bastar | -2.799 | 0.100 | -6.140 | 0.541 | 3.373 | 0.197 | -1.764 | 8.509 |
| **Age in years** | -0.027 | 0.715 | -0.173 | 0.119 | 0.012 | 0.887 | -0.151 | 0.175 |
| **Marital Status (Reference category - Married)** |  |  |  |  |  |  |  |  |
| Not married | -3.005 | 0.481 | -11.379 | 5.370 | 2.192 | 0.599 | -5.987 | 10.372 |
| **Household size** | -0.272 | 0.150 | -0.642 | 0.099 | -0.329 | 0.189 | -0.820 | 0.163 |
| **Social group of Mitanin (Reference category - vulnerable groups i.e. scheduled castes and tribes)** |  |  |  |  |  |  |  |  |
| Other Backward Classes | -0.219 | 0.826 | -2.173 | 1.736 | -0.373 | 0.792 | -3.159 | 2.412 |
| Others | 1.848 | 0.358 | -2.098 | 5.795 | 0.678 | 0.714 | -2.950 | 4.305 |
| **Education (Reference category - 8th standard or higher qualification)** |  |  |  |  |  |  |  |  |
| 5-7^th^ standard | 0.657 | 0.642 | -2.113 | 3.427 | -0.897 | 0.547 | -4.831 | 3.037 |
| 1-4^th^ standard | 0.856 | 0.758 | -4.601 | 6.312 | 0.554 | 0.631 | -3.331 | 4.44 |
| No formal education | 1.225 | 0.710 | -4.632 | 7.082 | -0.074 | 0.891 | -13.092 | 12.943 |

*p<0.05
