# Supplementary material for: Assessing the time use and payments of multipurpose community health workers for the various roles they play—a quantitative study of the Mitanin programme in India
Source: BMC Health Serv Res. 2022 Aug 10;22:1018. doi: 10.1186/s12913-022-08424-1 (PMC9364297; doi:10.1186/s12913-022-08424-1)
Supplement: Supplementary file 3 — Additional file 3. [file 12913_2022_8424_MOESM3_ESM.docx]

**Additional File S3- Activity wise distribution of average time spent by Mitanins in a week**

**Table S3.1 - Activity wise distribution of average time spent by rural Mitanins in a week (n=660)**

| **Purpose** | **Paid activities** | **Mean weekly time spent (hours)**  **A** | **Unpaid activities** | **Mean weekly time spent (hours)**  **B** | **% of time spent on unpaid tasks**  **B/(A+B)** |
| --- | --- | --- | --- | --- | --- |
| Health Education | Home visits for health education - newborn care and young child care (0-15 month age children) | 1.5 | Home visits for health education - young child care (16-35 month children) | 1.1 |  |
|  |  |  | Home visits for health education - pregnant women | 0.2 |  |
|  |  |  | Home visits for follow-up of chronic disease cases - e.g. hypertension, diabetes, mental disorders, blood disorders | 0.2 |  |
|  |  |  | Home visits for prevention of vector-borne and water-borne diseases e.g. dengue, malaria, diarrhoea | 0.2 |  |
|  |  |  | School health meetings with students | 0.2 |  |
|  |  |  | Meetings in community for health education on a wide range of health issues | 0.9 |  |
|  | **Sub-total** | **1.5** |  | **2.8** | **64%** |
| Delivering services directly | Testing and treatment of malaria | 0.5 | Identification and treatment of illnesses at Mitanin's home or by visiting families - diarrhoea, cold and cough in children, pneumonia in children, sick newborn, skin ailments, pain, minor injuries, reproductive-tract infections, eye infection | 1.5 |  |
|  |  |  | Contraceptive distribution and counselling | 0.1 |  |
|  | **Sub-total** | **0.5** |  | **1.6** | **76%** |
| Linkage with formal services | Attending monthly immunisation session/village health and nutrition day (classified as a monthly routine task) and bringing children for immunisation | 0.9 |  | 0.0 |  |
|  | Bringing pregnant women to local ante-natal care session, | 0.4 | Identification of high risk pregnancies, taking pregnant women to secondary care facilities for ante-natal check-ups, diagnostics and management | 0.5 |  |
|  | Identification of referrals and accompanying them to health facilities - deliveries, sterilisation, cataract, severely malnourished children | 1.0 | Identification of referrals or accompanying them to higher health facilities - sick newborn, pneumonia, non-communicable diseases, mental illnesses, injuries or other illnesses | 0.2 |  |
|  | Identification of presumptive cases of tuberculosis, leprosy; referring them to health facilities for confirmation; follow-ups for treatment adherence | 0.5 | Referrals for IUCD (other than PPIUCD) | 0.1 |  |
|  | Specific campaigns for linkage with services - pulse polio, de-worming, filaria prophylaxis, | 0.8 | Campaign for Vitamin A supplementation | 0.1 |  |
|  | Bringing patients especially above 30 years age individuals to health and wellness centres for screening of non-communicable diseases | 0.8 |  |  |  |
|  | **Sub-total** | **4.2** |  | **0.8** | **16%** |
| Action on social determinants of health (SDOH) | One monthly meeting of village health nutrition sanitation committee - for action on social determinants (classified as a monthly routine task) | 1.0 | Other community meetings for action on social determinants | 1.5 |  |
|  |  |  | Visits to officials of non-health sectors (water, food, employment etc.) | 0.1 |  |
|  |  |  | Home visits, community meetings and action on opposing gender based violence | 0.3 |  |
|  | **Sub-total** | **1.0** |  | **1.9** | **65%** |
| COVID-19 related action* |  |  | Home visits for COVID-19 related tasks-door to door survey to find persons with influenza symptoms, monitoring home isolation | 1.5 |  |
|  |  |  | Bringing individuals for testing, bringing individuals for COVID-19vaccination | 1.5 |  |
|  |  |  | Data collection and reporting related to COVID-19 | 0.7 |  |
|  | **Sub-total** | **0.0** |  | **3.7** | **100%** |
| Recording and reporting | Collecting data on population, symptoms and risk factors of multiple diseases in individuals using community based assessment checklist | 0.6 | Other surveys asked by government health officials | 0.6 |  |
|  | One monthly meeting for reporting to supervisors (classified as a monthly routine task) | 2.4 | Other meetings for reporting or for receiving instructions about activities for specific government campaigns and other priorities | 1.8 |  |
|  | Recording data in CHW register and updating monthly (classified as a monthly routine task) | 0.6 |  |  |  |
|  | **Sub-total** | **3.6** |  | **2.4** | **40%** |
|  | **Total** | **10.8** |  | **13.2** | **55%** |

**Table S3.2 - Activity wise distribution of average time spent by urban Mitanins in a week (n=406)**

| **Purpose** | **Paid activities** | **Mean weekly time spent (hours)**  **A** | **Unpaid activities** | **Mean weekly time spent (hours)**  **B** | **% of time spent on unpaid tasks**  **B/(A+B)** |
| --- | --- | --- | --- | --- | --- |
| Health Education | Home visits for health education - newborn care and young child care (0-15 month age children) | 1.9 | Home visits for health education - young child care (16-35 month children) | 1.2 |  |
|  |  |  | Home visits for health education - pregnant women | 0.3 |  |
|  |  |  | Home visits for follow-up of chronic disease cases - e.g. hypertension, diabetes, mental disorders, blood disorders | 0.3 |  |
|  |  |  | Home visits for prevention of vector-borne and water-borne diseases e.g. dengue, malaria, diarrhoea | 0.3 |  |
|  |  |  | School health meetings with students | 0.3 |  |
|  |  |  | Meetings in community for health education on a wide range of health issues | 1.0 |  |
|  | **Sub-total** | **1.9** |  | **3.2** | **62%** |
| Delivering services directly | Testing and treatment of malaria | 0.1 | Identification and treatment of illnesses at Mitanin's home or by visiting families - diarrhoea, cold and cough in children, pneumonia in children, sick newborn, skin ailments, pain, minor injuries, reproductive-tract infections, eye infection | 1.4 |  |
|  |  |  | Contraceptive distribution and counselling | 0.1 |  |
|  | **Sub-total** | **0.1** |  | **1.5** | **96%** |
| Linkage with formal services | Attending monthly immunisation session/village health and nutrition day (classified as a monthly routine task) and bringing children for immunisation | 2.0 |  |  |  |
|  | Bringing pregnant women to local ante-natal care session, | 0.7 | Identification of high risk pregnancies, taking pregnant women to secondary care facilities for ante-natal check-ups, diagnostics and management | 3.6 |  |
|  | Identification of referrals and accompanying them to health facilities - deliveries, sterilisation, cataract, severely malnourished children | 2.4 | Identification of referrals or accompanying them to higher health facilities - sick newborn, pneumonia, non-communicable diseases, mental illnesses, injuries or other illnesses | 0.7 |  |
|  | Identification of presumptive cases of tuberculosis, leprosy; referring them to health facilities for confirmation; follow-ups for treatment adherence | 1.4 | Referrals for IUCD (other than PPIUCD) | 0.1 |  |
|  | Specific campaigns for linkage with services - pulse polio, de-worming, filaria prophylaxis, | 1.1 | Campaign for Vitamin A supplementation | 0.1 |  |
|  | Bringing patients especially above 30 years age individuals to health and wellness centres for screening of non-communicable diseases | 1.4 |  |  |  |
|  | **Sub-total** | **8.9** |  | **4.6** | **34%** |
| Action on social determinants of health (SDOH) | One monthly meeting of village health nutrition sanitation committee - for action on social determinants (classified as a monthly routine task) | 1.0 | Other community meetings for action on social determinants | 1.4 |  |
|  |  |  | Visits to officials of non-health sectors (water, food, employment etc.) | 0.1 |  |
|  |  |  | Home visits, community meetings and action on opposing gender based violence | 0.3 |  |
|  | **Sub-total** | **1.0** |  | **1.8** | **64%** |
| COVID-19 related action* |  |  | Home visits for COVID-19 related tasks-door to door survey to find persons with influenza symptoms, monitoring home isolation | 1.5 |  |
|  |  |  | Bringing individuals for testing, bringing individuals for COVID-19vaccination | 1.5 |  |
|  |  |  | Data collection and reporting related to COVID-19 | 0.8 |  |
|  | **Sub-total** | **0.0** |  | **3.8** | **100%** |
| Recording and reporting | Collecting data on population, symptoms and risk factors of multiple diseases in individuals using community based assessment checklist | 0.7 | Other surveys asked by government health officials | 0.7 |  |
|  | One monthly meeting for reporting to supervisors (classified as a monthly routine task) | 2.8 | Other meetings for reporting or for receiving instructions about activities for specific government campaigns and other priorities | 2.1 |  |
|  | Recording data in CHW register and updating monthly (classified as a monthly routine task) | 0.7 |  |  |  |
|  | **Sub-total** | **4.1** |  | **2.8** | **38%** |
|  | **Total** | **16.0** |  | **17.6** | **52%** |
